# Microbiota and Metabolite Profiles in the Feces of Juvenile Sika Deer (*Cervus nippon*) from Birth to Weaning

**DOI:** 10.3390/ani14030432

**Published:** 2024-01-29

**Authors:** Ruina Mu, Songze Li, Yunxi Zhang, Yuqian Li, Yuhang Zhu, Fei Zhao, Huazhe Si, Zhipeng Li

**Affiliations:** 1 Joint International Research Laboratory of Modern Agricultural Technology, Ministry of Education, Jilin Agricultural University, Changchun 130118, China; 2 College of Animal Science and Technology, Jilin Agricultural University, Changchun 130118, China; 3 Jilin Provincial Engineering Research Center for Efficient Breeding and Product Development of Sika Deer, Jilin Agricultural University, Changchun 130118, China; 4 Key Laboratory of Animal Production, Product Quality and Security, Ministry of Education, Jilin Agricultural University, Changchun 130118, China

**Keywords:** juvenile sika deer, gut development, *Lactobacillus*, amino acid metabolism

## Abstract

**Simple Summary:**

The gut microbiota plays an active role in gut development and host growth in juvenile ruminants and contributes to their productive performance in adulthood. However, the gut microbiota composition and metabolic profile patterns of succession from birth to weaning of juvenile sika deer remain unclear. Feces are a highly efficient and convenient biological sample that can be used to reflect gut microbiology and metabolomics studies. Based on fecal samples, we demonstrated that the gut bacterial community and metabolic profile of juvenile sika deer were significantly altered at birth compared to the transition and rumination periods, suggesting functional changes in amino acid metabolism and carbohydrate metabolism. Our results revealed the key role of the birth–transition period in the regulation of gut bacterial communities and metabolic functions during juvenile sika deer development.

**Abstract:**

The gut microbiota establishment in young ruminants has a profound impact on their adult production performance. However, the critical phase for the succession of the gut microbial composition and metabolic profiles of juvenile sika deer still needs to be further investigated. Here, we analyzed the fecal microbiota and metabolites of juvenile sika deer during the birth (D1), transition (D42), and rumination (D70) periods based on 16S rRNA sequencing and gas chromatography–time–of–flight mass spectrometry (GC–TOF–MS). The results showed that the fecal bacteria and metabolites composition were significantly different in D1 compared to D42 and D70, and the number of OTUs and the Shannon index were significantly higher in D70 than in D1 (*p* < 0.05). The relative abundances of *Lactobacillus*, *Lactococcus*, and *Lachnoclostridium* showed a significant increase in D1 compared to D42 and D70, whereas the relative abundances of Ruminococcaceae UCG-005, Ruminococcaceae UCG-010, Ruminococcaceae UCG-014, Christensenellaceae R-7, and *Eubacterium coprostanoligenes* group were significantly decreased in D1 compared to D42 and D70 (*p* < 0.05). The amounts of serine, phenylalanine, aspartic acid, ornithine, citrulline, creatine, isoleucine, galactose, and ribose in the feces were significantly higher in D1 compared to D42 and D70. In contrast, the concentrations of cortexolone, resveratrol, piceatannol, fumaric acid, alpha-ketoglutarate, glycerol, uracil-5-carboxylic acid, and maleic acid were significantly decreased in D1. The enrichment analysis showed that amino acid metabolism and carbohydrate metabolism were significantly changed in D1 compared to D42 and D70. The glycine, serine and threonine metabolism; alanine, aspartate and glutamate metabolism; arginine biosynthesis; glyoxylate and dicarboxylate metabolism; citrate cycle; and pyruvate metabolism were significantly enriched across the three periods (*p* < 0.05). In conclusion, our results suggested that the birth–transition period is a critical phase for the gut bacterial community and metabolic function shift in juvenile sika deer.

## 1. Introduction

The gut microbiota is critical for maintaining nutrient metabolism and functions in ruminants [1]. There has been increasing interest in the bacterial communities that succeed in the intestinal tract of juvenile ruminant species due to the influence of colonization and succession in juvenile ruminants on the intestinal digestive and absorptive capacity in adulthood [2,3,4]. Although several studies have described the dynamic succession of the bacterial community and changes in metabolic functions in the gut of juvenile ruminants [5,6], the critical periods of community succession in the gut and the patterns of metabolic function shifts have not been fully explored. Thus, exploration of bacterial community succession in ruminants from birth to weaning, and whether the pattern of metabolic function changes, is critical for manipulating gut health.

Sika deer are farmed for more than one million heads in China for the production of velvet antlers, a traditional Chinese medicine [7]. The composition and community of gut bacteria have been found to play an important role in antler production [8], and the juvenile stage is regarded as a critical period for the regulation of gut microbial colonization [9]. Several studies have shown that the development of the gastrointestinal tract (GIT) from birth to weaning in sika deer has been categorized into three states: the non-rumination phase (0–21 days), transition phase (21–56 days), and rumination phase (from 56 days onwards) [10]. Unfortunately, limited information is known about the key stages of the changes in the gut microbial composition and metabolic functions in the gut of juvenile sika deer, although the farmed juvenile sika deer is weaned at about 2 months. We attempted to investigate the microbiota colonization and the metabolites composition in the small and large intestines of sika deer and demonstrated that the abundance of bacteria regulating the host immune function and the concentrations of short-chain fatty acids (SCFAs) were increased, respectively [11,12,13]. However, the gut is continuous, and metabolites produced in the small intestine are probably reutilized by microorganisms in the large intestinal fraction [14]. Thus, understanding the changes in the microbial succession and functions of the whole gut [15] could contribute to identifying the key metabolites that could be used to regulate growth and metabolism during juvenile sika deer farming. Feces, distinct from the contents of the gut, are usually used to represent the overall metabolic functions and microbial composition of the animal gut [16]. Previous studies have demonstrated that changes in fecal microbiota could influence the intestinal metabolic functions and immune status in calves and lambs [17,18]. Kim et al. (2021) indicated that fecal microbial transplantation could improve diarrhea and enhance the growth performance in pre-weaned calves [19]. Moreover, Yin et al. (2023) showed that the microbial composition of feces provides a reflection of the composition and function of intestinal bacteria in lambs [20]. These findings together suggest that it is efficient and convenient to use fecal samples to understand the microbiota succession and metabolic function shifts in juvenile sika deer.

In the present study, we analyzed the fecal microbiota and metabolites of juvenile sika deer during birth (D1), transition (D42), and rumination (D70) using 16S rRNA sequencing and gas chromatography–time–of–flight mass spectrometry (GC–TOF–MS), and explored the pattern of bacterial community succession and the changes in the metabolic functions in the gut, which provided insights into understanding the interactions between the gut microbiota and metabolism, and the regulation of juvenile deer growth and development.

## 2. Materials and Methods

### 2.1. Experimental Animals and Sample Collection

In this study, five healthy juvenile sika deer (female = 3, male = 2) of similar birth times and weights were selected from the Jilin Agricultural University research farm. These five young deer were kept with their dams in the same pen. After 60 days of the experiment, these five young deer were separated from their dams and provided with corn silage and concentrate diets. Clean water was freely available to all the animals throughout the study. Cryogenic storage tubes were used for collecting the feces on day 1, 42, and 70 of the experiment. For the microbiome and metabolome analysis, the samples were frozen directly in liquid nitrogen and stored at −80 °C. All the animal-specific procedures were approved and authorized by the Animal Ethics Committee of Jilin Agricultural University.

### 2.2. Extracting DNA, High-Throughput Sequencing and Sequences Analysis

A QIAamp^®^ Fast DNA Stool Mini Kit (QIAGEN, Valencia, CA, USA) was used to extract microbiological genomic DNA from the feces. The amplification of the V3 to V4 region of the 16S rRNA gene of bacteria was achieved using primers (341F and 806R) [21]. A QIAquick PCR Purification Kit (QIAGEN, Valencia, CA, USA) was used to purify the amplicons. A paired 250-bp read was generated by combining the PhiX Control library (Illumina, 20%) with the amplicon library, then normalizing and sequencing them on the Illumina PE MiSeq platform.

### 2.3. Profiling the Metabolites of the Fecal Samples

The metabolites in the feces were analyzed using gas chromatography–time–of–flight mass spectrometry (GC–TOF–MS), according to the reported procedure [22] and methods [11]. The Chroma TOF software (version 5.0) from LECO Corporation and the LECO-Fiehn Rtx5 database were employed for peak picking, data baseline filtering and calibration of the baseline, peak alignment, peak identification, and integration of the peak area [23]. The metabolites with a similarity criterion of less than 300 were removed from the LECO/Fiehn metabolomics library.

### 2.4. Bioinformatics and Statistical Analyses

Contiguous sequences were performed for primer deletion and quality control using FLASH [24], and then the obtained sequences were imported into QIIME v1.9.1 [25]. Based on a sequence similarity of 97%, the sequences were clustered into operational taxonomic units (OTUs) using UPARSE [26], after removing potential chimeric sequences using UCHIME [27]. Representative sequences of the OTUs were classified using the RDP classifier and annotated according to the SILVA database (version 123) [28]. The alpha diversity indices, including the OTUs, Chao1, and Shannon indices, were subsequently calculated using the R microeco package [29]. The Tax4Fun [30] was applied to predict the functional profiles of the fecal microbiota based on the Silva reference database (version 123). A principal coordinate analysis (PCoA) was used to compare the bacterial communities and functions in the feces of sika deer among the three age groups based on the weighted UniFrac distance, unweighted UniFrac distance, and Bray–Curtis dissimilarity. Furthermore, the group similarity was calculated via an analysis of similarities (ANOSIM) and a permutational multivariate analysis of variance (Adonis).

The principal component analysis (PCA) of the fecal metabolites was calculated using the prcomp function of the stats package and ropls package [31]. The omicade4 package was utilized for multiple co-inertia analysis (MCIA) of the microbiota and metabolites [32]. The stats package was used to calculate Spearman’s rank correlation coefficient with a threshold of R > 0.6 or <−0.6 and *p* < 0.05, and Cytoscape (version 3.9.1) was applied to visualize the association networks [33].

The differences in the alpha diversity indices and fecal metabolites among the three age groups were determined using the Kruskal–Wallis (KW) test, and the differences in the fecal microbiota and functions were performed using the linear discriminant analysis effect size (LefSe) method [34]. The false discovery rate of the Benjamini–Hochberg method was used to correct the *p* values of the KW tests. A value of *p* ≤ 0.05 was considered to indicate a statistical significance.

## 3. Results

### 3.1. Taxonomic Composition of the Fecal Microbiota

**Figure 1:**
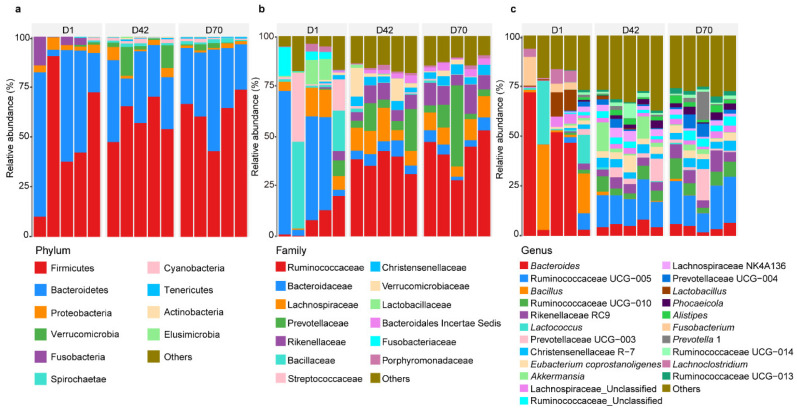
Bacterial community composition in the feces of juvenile sika deer in the three age groups. Bacterial profiles at the phylum level (**a**), family level (**b**), and genus level (**c**). D1 = day 1, D42 = day 42, D70 = day 70.

In this study, a total of 459,494 sequences with a median length of 411 bp (401–450 bp) were assigned to 1334 OTUs from all the samples. Based on these OTUs, we identified a total of 15 bacterial phyla (present in at least one sample) in the feces (Figure 1a). Firmicutes was the dominant phylum in the feces in D1 (50.43%), D42 (58.61%), and D70 (61.39%), followed by Bacteroidetes (D1 = 40.27%, D42 = 28.47%, D70 = 32.62%). At the family level, a total of 106 families were identified in the feces (Figure 1b). Bacteroidaceae (35.22%) was the most prevalent bacteria in the feces of sika deer in D1, followed by Bacillaceae (13.33%) and Streptococcaceae (10.55%), which accounted for 59.10% of the total bacteria. In D42 and D70, Ruminococcaceae (37.40% and 42.75%), Prevotellaceae (9.38% and 12.32%), and Lachnospiraceae (10.47% and 8.71%) were the dominant bacteria, accounting for 64.07% and 73.31% of the total bacteria, respectively.

Furthermore, a total of 249 genera were detected in the feces of sika deer in the three age groups (Figure 1c). *Bacteroides* (35.22%), *Bacillus* (13.17%), and *Lactococcus* (9.70%) were predominant in D1, while Ruminococcaceae UCG-005, Ruminococcaceae UCG-010, and Rikenellaceae RC9 were prevalent in D42 (15.35%, 5.11%, and 4.40%, respectively) and D70 (18.23%, 6.60%, and 6.51%, respectively).

### 3.2. Microbial Diversity and Communities of Feces among the Three Age Groups

**Figure 2:**
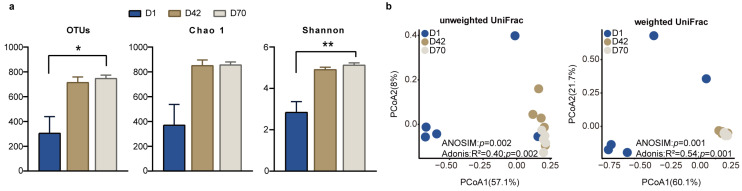
Comparison of the diversity of the fecal microbiota in the juvenile sika deer among the three age groups. (**a**) Alpha diversity indices in the feces of juvenile sika deer among D1, D42, and D70. * *p* < 0.05, ** *p* < 0.01. (**b**) The PCoA shows the separation of the fecal bacterial community among the three age groups at the OTU level based on the unweighted UniFrac distance and weighted UniFrac distance.

We further determined the difference in the fecal bacterial community among the three age groups. Comparisons of the diversity indices showed that the number of OTUs and the Shannon index were significantly higher in D70 than in D1 (*p* < 0.05), while the Chao 1 index was not significantly different among the three age groups (*p* > 0.05; Figure 2a). The PCoA results based on the unweighted UniFrac distance and weighted UniFrac distance showed that the fecal bacterial community and composition in D1 were clearly separated from those in D42 and D70 (*p* < 0.05), and that the fecal bacterial community and composition in D42 were clustered together with those in D70 (Figure 2b).

### 3.3. Microbial Abundance and Function Changes among the Three Age Groups

**Figure 3:**
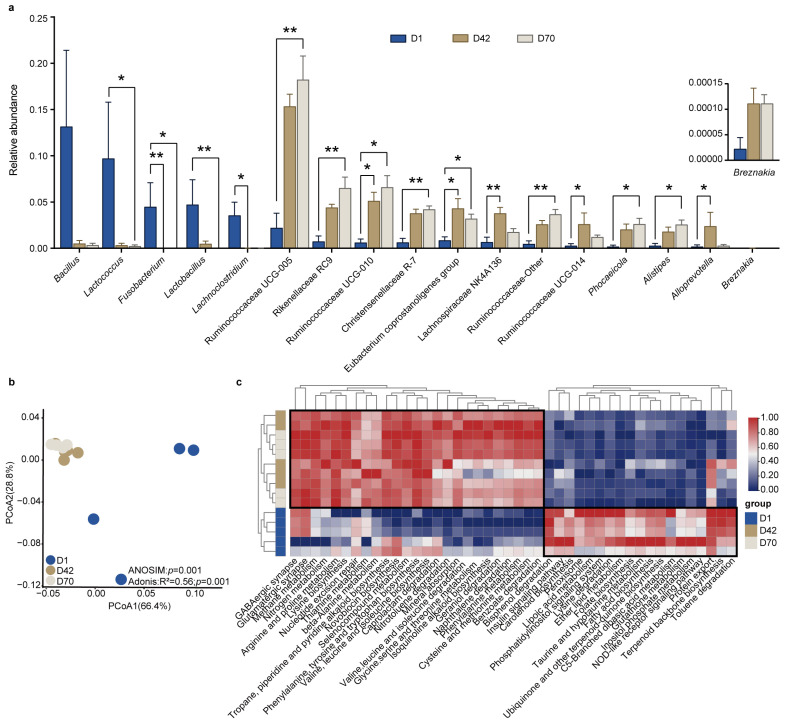
Comparison of the significantly different bacterial genera and functions in the feces of juvenile sika deer among the different age groups. (**a**) The bar plot shows the significantly different bacterial genera in the feces of juvenile sika deer among the different age groups using the LEfSe based on the KW test (LDA > 4, *p* < 0.05). * *p* < 0.05, ** *p* < 0.01. (**b**) The PCoA shows the difference in the bacterial functional profiles at KEGG level 3 (relative abundances) based on the Bray–Curits dissimilarity. (**c**) Heatmap showing the significantly changed pathways at KEGG level 3 among the different age groups (LDA > 2, *p* < 0.05).

We next examined the significantly different bacterial genera among the three age groups (Figure 3a and Table S1). The results showed that the relative abundances of *Lactobacillus*, *Lactococcus*, *Lachnoclostridium*, and *Fusobacterium* were significantly higher in D1 than in D42 and D70. However, the relative abundances of *Alistipes*, *Phocaeicola*, Christensenellaceae R-7, Rikenellaceae RC9, Ruminococcaceae Other, Ruminococcaceae UCG-005, and Ruminococcaceae UCG- 010 were higher in D70 compared to D1 and D42. In addition, *Alloprevotella*, Lachnospiraceae NK4A136, *Eubacterium coprostanoligenes* group, and Ruminococcaceae UCG-014 were significantly higher in D42 than D1 and D70 (LDA > 4, *p* < 0.05).

We then applied Tax4Fun to predict the potential functions of the fecal bacteria and compared the differences among the three age groups. The PCoA results showed that the metabolic pathways at KEGG level 3 in D1 were clearly separated from those in D42 and D70 (*p* < 0.05; Figure 3b). Moreover, a total of 44 pathways were significantly different among D1, D42, and D70 (LDA > 2, *p* < 0.05). The relative abundances of amino acid metabolism, including arginine and proline metabolism; phenylalanine metabolism; cysteine and methionine metabolism; glycine, serine and threonine metabolism; valine, leucine and isoleucine biosynthesis; phenylalanine, tyrosine and tryptophan biosynthesis; and lysine biosynthesis were significantly increased in D42 and D70 compared to those in D1. However, the pathways of carbohydrate and lipid metabolism, including galactose metabolism, glycolysis/gluconeogenesis, inositol phosphate metabolism, pentose phosphate pathway, glycerophospholipid metabolism, and fatty acid biosynthesis were significantly decreased in D42 and D70 compared to those in D1 (*p* < 0.05; Figure 3c).

### 3.4. Changes in the Fecal Metabolites among the Three Age Groups

**Figure 4:**
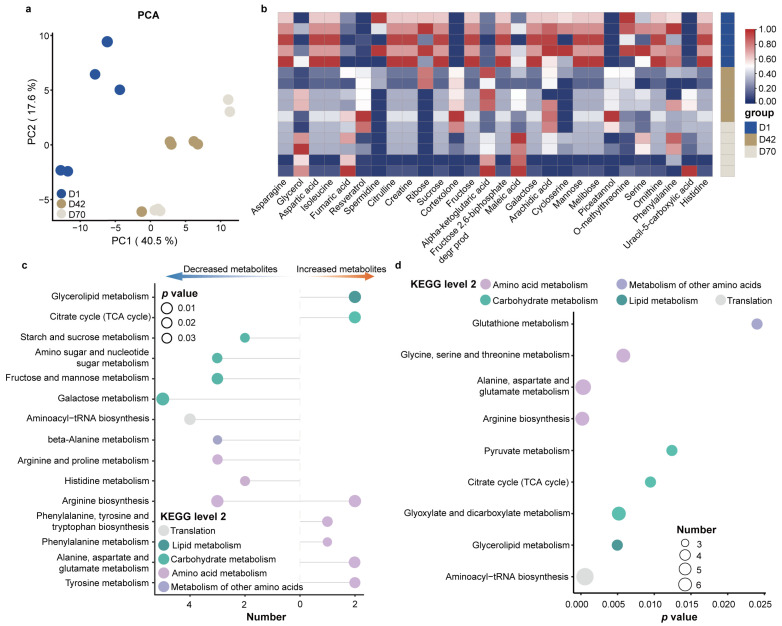
Fecal metabolic profiles of sika deer from birth to weaning. (**a**) PCA of the fecal metabolites of sika deer. (**b**) Heatmap showing the significantly different metabolites among the different age groups. Metabolic pathway enrichment analysis based on significantly different metabolites (**c**) and shared metabolites (**d**) among the three age groups (*p* < 0.05).

We further examined the fecal metabolites, and a total of 137 metabolites were identified in the feces of juvenile sika deer, mainly including fatty acids, amino acids, amines, organic acids, purines, pyrimidines, sugars, and sugar alcohols (Table S2). The PCA results showed a clear separation between D42, D70 and D1 (Figure 4a). Subsequently, we identified a total of 28 metabolites that were significantly different in the feces (Figure 4b). The concentrations of serine, phenylalanine, aspartic acid, ornithine, citrulline, creatine, isoleucine, galactose, and ribose in the fecal metabolites were higher in D1 compared to D42 and D70. In contrast, cortexolone, resveratrol, piceatannol, fumaric acid, alpha-ketoglutarate, glycerol, uracil-5-carboxylic acid, and maleic acid were greater in D42 and D70 (*p* < 0.05). We further applied the metabolic pathways enriched analysis to illustrate the difference in gut metabolic function between D42, D70 and D1 (Figure 4c). Compared to D1, the increased metabolites in D42 and D70 were significantly enriched in phenylalanine, tyrosine and tryptophan biosynthesis; arginine biosynthesis; alanine, aspartate and glutamate metabolism; phenylalanine metabolism; and tyrosine metabolism. In contrast, the decreased metabolites were significantly enriched in fructose and mannose metabolism, amino sugar and nucleotide sugar metabolism, starch and sucrose metabolism, and galactose metabolism (*p* < 0.05).

To further characterize the core metabolic functions of the gut microbiota during young deer development, we performed a metabolic pathway enrichment analysis based on the shared metabolites among the three age groups (Figure 4d and Table S3). The results showed that amino acid metabolism, including glycine, serine and threonine metabolism; alanine, aspartate and glutamate metabolism; and arginine biosynthesis, as well as carbohydrate metabolism, including glyoxylate and dicarboxylate metabolism, citrate cycle (TCA cycle), and pyruvate metabolism were significantly enriched (*p* < 0.05).

### 3.5. Correlation Analysis between the Fecal Microbiota and Metabolites

**Figure 5:**
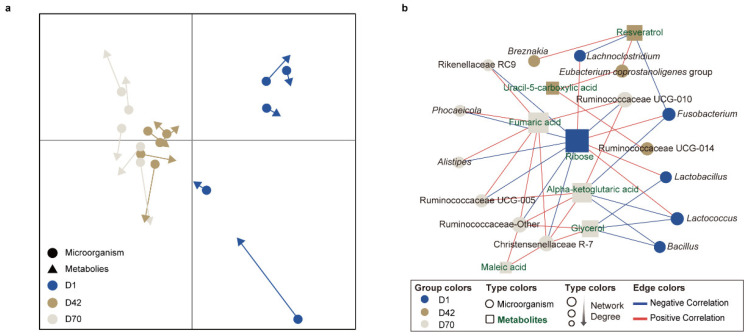
Associations of the significantly different fecal genera and metabolites in juvenile sika deer with different age groups. (**a**) MCIA of fecal bacterial functions among the different age groups. (**b**) Differences in the co-occurrence association networks of juvenile sika deer among the different age groups. The association network was visualized in Cytoscape using the radial layout algorithm. The circles and rectangles represent microorganisms and metabolites, respectively. The colors in the notes indicate the bacterial genera or metabolites with the highest relative abundances or concentrations in D1, D42, and D70, respectively. The blue and red edge lines represent the negative and positive correlations, respectively.

To identify the relationships between the fecal microbiota and metabolites among the different age groups of sika deer, we performed MCIA using the microbiotas and metabolomics datasets and observed a remarkable separation between D42, D70 and D1 (Figure 5a). The Spearman correlation analysis was applied to determine the potential correlations between the differentially altered bacteria and metabolites among the three age groups, and an integrated network was identified and represented (R > 0.6 or <−0.6, *p* < 0.05, Figure 5b). The results showed two visually highlighted clusters of bacteria around ribose and fumaric acid. Among them, ribose was positively correlated with *Lactobacillus*, *Lactococcus*, *Lachnoclostridium*, and *Fusobacterium* and negatively correlated with *Alistipes*, Christensenellaceae R-7, Rikenellaceae RC9, *Phocaeicola*, Ruminococcaceae UCG-005, Ruminococcaceae UCG-010, and Ruminococcaceae Other. Fumaric acid was positively correlated with Ruminococcaceae UCG-005, Ruminococcaceae UCG-010, Ruminococcaceae Other, *Alistipes*, Rikenellaceae RC9, *Phocaeicola* and Christensenellaceae R-7. Moreover, both fumaric acid, maleate acid, and alpha-ketoglutaric acid showed positive correlations with the family Ruminocococcaceare and Christensenellaceae R-7 (R > 0.60, *p* < 0.05).

## 4. Discussion

In this study, we analyzed the shifts in the fecal microbiota and metabolites in sika deer from birth to weaning based on 16S rRNA sequencing and GC-TOF-MS. The results showed that Bacteroidetes and Firmicutes were predominant in the feces across three stages, consistent with the findings in yaks, calves, and goats [35,36]. This finding highlights the central role of Firmicutes and Bacteroidetes in the gut development of sika deer [37]. Previous studies have shown that an increased ratio of Firmicutes to Bacteroidetes (FB ratio) can promote intestinal nutrient absorption and circulation [38,39]. In this study, we found that the FB ratio increased with age (1.25 to 1.88), suggesting increased metabolism and nutrient absorption with the growth of sika deer.

*Bacteroides*, *Bacillus*, and *Lactococcus* were predominant in D1, and Ruminococcaceae UCG-005, Ruminococcaceae UCG-010, and Rikenellaceae RC9 were predominant in D42 and D70. The bacteria detected in the intestinal tract of young ruminants in calves and sheep were mainly *Bacillus* and *Lactococcus* [40,41] since they are usually present in breast milk or in the birth canal. Ruminococcaceae UCG-005, Ruminococcaceae UCG-010, and Rikenellaceae RC9, with the functions of degrading cellulose and starch, were also widely present in the intestinal tracts of young ruminants during the transitional ruminant periods [36,42]. These results suggested that the stage from birth to the transition period may be a critical stage for the succession of microbial communities in their gut. We also found that the diversity of fecal bacteria increased rapidly in D42, consistent with the findings in goats and calves [6,43], suggesting that solid diets can increase the diversity and abundance of gut microbiota in young ruminants. Similarly, the PCoA results also showed a clear separation between D42, D70 and D1. A similar trend was observed in the different digestive tract regions of juvenile deer [11,12,13]. These findings together demonstrated the presence of a unique bacterial community in the feces of sika deer at birth, followed by the establishment of a stable bacterial community during the transitional to ruminant periods.

The results revealed that the relative abundances of *Lactobacillus*, *Lactococcus*, *Fusobacterium*, and *Lachnoclostridium* were significantly higher during the birth period compared to the other periods. Previous studies showed that *Lactobacillus* and *Lactococcus lactis* can hydrolyze dietary lactose to lactic acid via lactase [44,45], while *Lachnoclostridium* and *Fusobacterium* can further metabolize lactic acid to produce SCFA [46,47], which provides energy for intestinal epithelial cell growth. Consistently, previous studies showed that *Lactobacillus* and *Lactococcus* were found in the milk of cows and in the feces of calves [48,49]. These results suggested that bacterial colonization at birth is likely related to dietary composition. In contrast, the relative abundances of *Alistipes*, Christensenellaceae R-7, Rikenellaceae RC9, Ruminococcaceae Other, Ruminococcaceae UCG-005, and Ruminococcaceae UCG- 010 increased linearly with age, which was in line with the findings for calves and goats [40,50]. As discussed above, these bacteria play important roles in carbohydrate and nitrogen degradation [51,52], which may reveal commonalities in the succession of gut microbial communities in juvenile ruminants. Previous studies have demonstrated that the supplementation of polysaccharides could increase the body weight of calves and reduce the rate of calf diarrhea by affecting the abundance of beneficial intestinal bacteria, thus promoting calf growth [53,54], indicating the possibility and importance of polysaccharides for promoting gut health and growth in juvenile sika deer.

After the establishment of the pioneer microbiota, the GIT undergoes a process of colonization from transitional microbial communities. This colonization process is influenced by bidirectional drivers, including intrinsic factors associated with the gastrointestinal tract development and extrinsic factors related to changes in dietary patterns [55]. In the present study, we found that the relative abundances of *Eubacterium coprostanoligenes* group, *Alloprevotella*, and Lachnospiraceae NK4A136 were significantly higher in D42 than in D1 and D70, suggesting that these bacteria play important roles in the transitional stage. *Eubacterium* plays a vital role in amino acid fermentation [56], and its abundance changes may provide evidence for revealing the relationship between the establishment of amino acid metabolism in the gut and the succession of microbial communities. Accordingly, the amino acid metabolism of fecal bacteria was significantly higher in both the transitional and ruminant stages compared to the birth stage, which was consistent with our results discussed above. These results suggested that in stable bacterial structures, changes in the microbial composition or abundance may have less impact on their function. Recent findings showed the assembly dynamics of the rumen microbiome of cows during the first 3 years of life and revealed the importance of the deterministic environmental factors and stochastic factors [57], suggesting that more samples from sika deer post-weaning to adulthood are needed. Moreover, the comparison of the predicted metabolic pathways showed that the relative abundances of carbohydrate metabolism (galactose metabolism, glycolysis/gluconeogenesis, inositol phosphate metabolism, and pentose phosphate pathway) in D70 and D42 were decreased compared to D1. One explanation for this result was that the rumen became the first site of carbohydrate fermentation after the development of the digestive tract in ruminants, thus affecting the availability of carbohydrates in the intestine [58]. However, future studies based on metagenomic analyses could further confirm the microbial activity in the feces of sika deer during early life.

The PCA results also showed a separation of fecal metabolites between D42, D70 and D1, which was similar to the change in the bacterial composition, revealing a common shift between gut microbial community succession and metabolite composition in young deer. The concentrations of ornithine, citrulline, and creatine were significantly higher in group D1 compared to D42 and D70, which were the intermediates or end products of arginine metabolism, suggesting that arginine metabolism may have been enriched at birth [59,60]. On the other hand, the significantly increased metabolites in D42 and D70 partly belonged to plant secondary metabolites (cortexolone, resveratrol, piceatannol, fumaric acid, and maleic acid), which may have been related to the composition of the diets during the transition and ruminant periods [61,62]. We also found that the amino acid metabolism and carbohydrate metabolism pathways were enriched with sika deer growth. The most common amino acid fermenters in the mammalian gut include members from the class Clostridia [63]. This was supported by the significantly increased bacteria, including Ruminococcaceae UCG-005, Ruminococcaceae UCG-010, Ruminococcaceae UCG-014, Ruminococcaceae Other, Christensenellaceae R-7, *Eubacterium coprostanoligenes* group, and Lachnospiraceae NK4A136 during the transitional and ruminant periods. These results suggested the enhanced ability of the utilization of solid feeds. Interestingly, we found that amino acid metabolism, including glycine, serine and threonine metabolism; alanine, aspartate and glutamate metabolism; and arginine biosynthesis, as well as carbohydrate metabolism, including glyoxylate and dicarboxylate metabolism and pyruvate metabolism, were significantly enriched based on the shared metabolites from birth to weaning. These findings suggested that these metabolic pathways play important roles in the gut development of juvenile sika deer.

The similarity between the microbiota and metabolites increased with age. Moreover, the predominant bacterial genera of feces in D70, including *Alistipes*, Christensenellaceae R-7, Rikenellaceae RC9, Ruminococcaceae UCG-005, and Ruminococcaceae UCG-010, were also indicated as dominant bacteria in the feces of adult deer [64,65,66], suggesting that the bacteria and metabolites gradually formed stable integrated systems in the feces of sika deer. Moreover, ribose and fumaric acid showed a positive correlation with the bacteria that were significantly increased in abundance in D1 or D70, respectively. Previous studies have demonstrated that ribose can serve as a fermentative carbon source for *Lactobacillus* [67], *Lactococcus* [68], and *Lachnoclostridium* [69], suggesting that ribose provides a nutritional substrate for the pioneer bacteria of the deer gut at birth. Moreover, fumaric acid, maleate acid, and alpha-ketoglutaric acid were both involved in butanoate metabolism and showed positive correlations to the family Ruminocococcaceare. Members of the bacteria belonging to Ruminocococcaceare play an important role in butyrate production [70]. This also suggests that butyrate metabolism in gut of sika deer may be further enhanced during the ruminant period.

## 5. Conclusions

In this study, we found significant changes in the fecal bacteria composition and communities during birth to rumination in young sika deer, with the birth–transition period being the critical time for the establishment of the gut microbiota. The metabolomics results indicated that the shift in gut metabolic function in juvenile deer was dominated by carbohydrate and amino acid metabolism. Our results also further suggested the potential role of arginine in the development of the gut in juvenile deer, providing evidence for the regulation of the gut microbiota and metabolic functions in sika deer.

## Data Availability

The data presented in this study are available upon request from the corresponding authors. The data are not publicly available due to privacy or ethical restrictions.

## Supplementary Materials

The following supporting information can be downloaded at: https://www.mdpi.com/article/10.3390/ani14030432/s1, Table S1: The significantly different bacterial genera (relative abundances) in the feces of juvenile sika deer among different age groups; Table S2: The classification and proportion of identified metabolites; Table S3: Metabolic pathway enrichment analysis based on the shared metabolites among the three age groups.

## References

[1] O’Hara E., Neves A.L.A., Song Y., Guan L.L. (2020). The role of the gut microbiome in cattle production and health: Driver or passenger?. Annu. Rev. Anim. Biosci..

[2] Du Y., Gao Y., Hu M., Hou J., Yang L., Wang X., Du W., Liu J., Xu Q. (2023). Colonization and development of the gut microbiome in calves. J. Anim. Sci. Biotechnol..

[3] He Z., Ma Y., Yang S., Zhang S., Liu S., Xiao J., Wang Y., Wang W., Yang H., Li S. (2022). Gut microbiota-derived ursodeoxycholic acid from neonatal dairy calves improves intestinal homeostasis and colitis to attenuate extended-spectrum β-lactamase-producing enteroaggregative *Escherichia coli* infection. Microbiome.

[4] Zhang K., Li B., Guo M., Liu G., Yang Y., Wang X., Chen Y., Zhang E. (2019). Maturation of the goat rumen microbiota involves three stages of microbial colonization. Animals.

[5] Zhang X., Wu J., Zhou C., Tan Z., Jiao J. (2021). Spatial and temporal organization of jejunal microbiota in goats during animal development process. J. Appl. Microbiol..

[6] Guo J., Li P., Zhang K., Zhang L., Wang X., Li L., Zhang H. (2020). Distinct stage changes in early-life colonization and acquisition of the gut microbiota and its correlations with volatile fatty acids in goat kids. Front. Microbiol..

[7] Wu F., Li H., Jin L., Li X., Ma Y., You J., Li S., Xu Y. (2013). Deer antler base as a traditional Chinese medicine: A review of its traditional uses, chemistry and pharmacology. J. Ethnopharmacol..

[8] Si H., Han Y., Liu H., Lou Y., Li Z. (2021). Effects of rumen-protected arginine supplementation on the plasma amino acids and gut microbiota of sika deer (*Cervus nippon*). Anim. Feed Sci. Technol..

[9] Abecia L., Martín-García A.I., Martínez G., Newbold C.J., Yáñez-Ruiz D.R. (2013). Nutritional intervention in early life to manipulate rumen microbial colonization and methane output by kid goats postweaning. J. Anim. Sci..

[10] Jiao J., Huang J., Zhou C., Tan Z. (2015). Taxonomic identification of ruminal epithelial bacterial diversity during rumen development in goats. Appl. Environ. Microbiol..

[11] Li Z., Wang X., Zhang T., Si H., Nan W., Xu C., Guan L., Wright A.G., Li G. (2018). The development of microbiota and metabolome in small intestine of sika deer (*Cervus nippon*) from birth to weaning. Front. Microbiol..

[12] Li Z., Si H., Nan W., Wang X., Zhang T., Li G. (2019). Bacterial community and metabolome shifts in the cecum and colon of captive sika deer (*Cervus nippon*) from birth to post weaning. FEMS Microbiol. Lett..

[13] Yan X., Si H., Zhu Y., Li S., Han Y., Liu H., Du R., Pope P.B., Qiu Q., Li Z. (2022). Integrated multi-omics of the gastrointestinal microbiome and ruminant host reveals metabolic adaptation underlying early life development. Microbiome.

[14] Abdallah A., Elemba E., Zhong Q., Sun Z. (2020). Gastrointestinal interaction between dietary amino acids and gut microbiota: With special emphasis on host nutrition. Curr. Protein Pept. Sci..

[15] Uchiyama J., Murakami H., Sato R., Mizukami K., Suzuki T., Shima A., Ishihara G., Sogawa K., Sakaguchi M. (2020). Examination of the fecal microbiota in dairy cows infected with bovine leukemia virus. Vet. Microbiol..

[16] Zierer J., Jackson M.A., Kastenmüller G., Mangino M., Long T., Telenti A., Mohney R.P., Small K.S., Bell J.T., Steves C.J. (2018). The fecal metabolome as a functional readout of the gut microbiome. Nat. Genet..

[17] Shi Z., Wang Y., Yan X., Ma X., Duan A., Hassan F.-u., Wang W., Deng T. (2023). Metagenomic and metabolomic analyses reveal the role of gut microbiome-associated metabolites in diarrhea calves. mSystems.

[18] Wang X., Niu L., Wang Y., Zhan S., Wang L., Dai D., Cao J., Guo J., Li L., Zhang H. (2023). Combining 16S rRNA sequencing and metabolomics data to decipher the interactions between gut microbiota, gost Immunity, and metabolites in diarrheic young small ruminants. Int. J. Mol. Sci..

[19] Kim H.S., Whon T.W., Sung H., Jeong Y.S., Jung E.S., Shin N.R., Hyun D.W., Kim P.S., Lee J.Y., Lee C.H. (2021). Longitudinal evaluation of fecal microbiota transplantation for ameliorating calf diarrhea and improving growth performance. Nat. Commun..

[20] Yin X.J., Ji S.K., Duan C.H., Tian P.Z., Ju S.S., Yan H., Zhang Y.J., Liu Y.Q. (2023). The succession of fecal bacterial community and its correlation with the changes of serum immune indicators in lambs from birth to 4 months. J. Integr. Agric..

[21] The Human Microbiome Project Consortium (2012). Structure, function and diversity of the healthy human microbiome. Nature.

[22] Sun H.Z., Wang D.M., Wang B., Wang J.K., Liu H.Y., le Guan L., Liu J.X. (2015). Metabolomics of four biofluids from dairy cows: Potential biomarkers for milk production and quality. J. Proteome Res..

[23] Kind T., Wohlgemuth G., Lee D.Y., Lu Y., Palazoglu M., Shahbaz S., Fiehn O. (2009). FiehnLib: Mass spectral and retention index libraries for metabolomics based on quadrupole and time-of-flight gas chromatography/mass spectrometry. Anal. Chem..

[24] Magoč T., Salzberg S.L. (2011). FLASH: Fast length adjustment of short reads to improve genome assemblies. Bioinformatics.

[25] Caporaso J.G., Kuczynski J., Stombaugh J., Bittinger K., Bushman F.D., Costello E.K., Fierer N., Peña A.G., Goodrich J.K., Gordon J.I. (2010). QIIME allows analysis of high-throughput community sequencing data. Nat. Methods.

[26] Edgar R.C. (2013). UPARSE: Highly accurate OTU sequences from microbial amplicon reads. Nat. Methods.

[27] Edgar R.C., Haas B.J., Clemente J.C., Quince C., Knight R. (2011). UCHIME improves sensitivity and speed of chimera detection. Bioinformatics.

[28] Henderson G., Yilmaz P., Kumar S., Forster R.J., Kelly W.J., Leahy S.C., Guan L.L., Janssen P.H. (2019). Improved taxonomic assignment of rumen bacterial 16S rRNA sequences using a revised SILVA taxonomic framework. PeerJ.

[29] Liu C., Cui Y., Li X., Yao M. (2021). Microeco: An R package for data mining in microbial community ecology. FEMS Microbiol. Ecol..

[30] Aßhauer K.P., Wemheuer B., Daniel R., Meinicke P. (2015). Tax4Fun: Predicting functional profiles from metagenomic 16S rRNA data. Bioinformatics.

[31] Thévenot E.A., Roux A., Xu Y., Ezan E., Junot C. (2015). Analysis of the human adult urinary metabolome variations with age, body mass index, and gender by implementing a comprehensive workflow for univariate and OPLS statistical analyses. J. Proteome Res..

[32] Meng C., Kuster B., Culhane A.C., Gholami A.M. (2014). A multivariate approach to the integration of multi-omics datasets. BMC Bioinform..

[33] Franz M., Lopes C.T., Huck G., Dong Y., Sumer O., Bader G.D. (2016). Cytoscape.js: A graph theory library for visualisation and analysis. Bioinformatics.

[34] Segata N., Izard J., Waldron L., Gevers D., Miropolsky L., Garrett W.S., Huttenhower C. (2011). Metagenomic biomarker discovery and explanation. Genome Biol..

[35] Zhang J., Liang Z., Ding Kao R., Han J., Du M., Ahmad A.A., Wang S., Salekdeh G.H., Long R., Yan P. (2022). Maternal fecal microbes contribute to shaping the early life assembly of the intestinal microbiota of co-inhabiting yak and cattle calves. Front. Microbiol..

[36] Liao R., Xie X., Lv Y., Dai J., Lin Y., Zhu L. (2021). Ages of weaning influence the gut microbiota diversity and function in Chongming white goats. Appl. Microbiol. Biotechnol..

[37] Hu X., Liu G., Li Y., Wei Y., Lin S., Liu S., Zheng Y., Hu D. (2018). High-throughput analysis reveals seasonal variation of the gut microbiota composition within forest musk deer (*Moschus berezovskii*). Front. Microbiol..

[38] Turnbaugh P.J., Ley R.E., Mahowald M.A., Magrini V., Mardis E.R., Gordon J.I. (2006). An obesity-associated gut microbiome with increased capacity for energy harvest. Nature.

[39] Li H., Li T., Beasley D.E., Heděnec P., Xiao Z., Zhang S., Li J., Lin Q., Li X. (2016). Diet diversity is associated with beta but not alpha diversity of pika gut microbiota. Front. Microbiol..

[40] Li B., Zhang K., Li C., Wang X., Chen Y., Yang Y. (2019). Characterization and comparison of microbiota in the gastrointestinal tracts of the goat (*Capra hircus*) during preweaning development. Front. Microbiol..

[41] Li K., Shi B., Na R. (2023). The colonization of rumen microbiota and intervention in pre-weaned ruminants. Animals.

[42] Song Y., Malmuthuge N., Steele M.A., Guan L.L. (2018). Shift of hindgut microbiota and microbial short chain fatty acids profiles in dairy calves from birth to pre-weaning. FEMS Microbiol. Ecol..

[43] Klein-Jöbstl D., Schornsteiner E., Mann E., Wagner M., Drillich M., Schmitz-Esser S. (2014). Pyrosequencing reveals diverse fecal microbiota in Simmental calves during early development. Front. Microbiol..

[44] Kim H.S., Gilliland S.E. (1983). *Lactobacillus acidophilus* as a dietary adjunct for milk to aid lactose digestion in humans. J. Dairy. Sci..

[45] Roy K., Anba J., Corthier G., Rigottier-Gois L., Monnet V., Mistou M.Y. (2008). Metabolic adaptation of *Lactococcus lactis* in the digestive tract: The example of response to lactose. J. Mol. Microbiol. Biotechnol..

[46] Feng Y., Stams A.J.M., de Vos W.M., Sánchez-Andrea I. (2017). Enrichment of sulfidogenic bacteria from the human intestinal tract. FEMS Microbiol. Lett..

[47] Tadepalli S., Narayanan S.K., Stewart G.C., Chengappa M.M., Nagaraja T.G. (2009). *Fusobacterium necrophorum*: A ruminal bacterium that invades liver to cause abscesses in cattle. Anaerobe.

[48] Zhu H., Yang M., Loor J.J., Elolimy A., Li L., Xu C., Wang W., Yin S., Qu Y. (2021). Analysis of cow-calf microbiome transfer routes and microbiome diversity in the newborn holstein dairy calf hindgut. Front. Nutr..

[49] Wei W., Hu X., Hou Z., Wang Y., Zhu L. (2021). Microbial community structure and diversity in different types of non-bovine milk. Curr. Opin. Food Sci..

[50] Amin N., Schwarzkopf S., Tröscher-Mußotter J., Camarinha-Silva A., Dänicke S., Huber K., Frahm J., Seifert J. (2023). Host metabolome and faecal microbiome shows potential interactions impacted by age and weaning times in calves. Anim. Microbiome.

[51] Ozbayram E.G., Ince O., Ince B., Harms H., Kleinsteuber S. (2018). Comparison of rumen and manure microbiomes and implications for the inoculation of anaerobic digesters. Microorganisms.

[52] Xin J., Chai Z., Zhang C., Zhang Q., Zhu Y., Cao H., Zhong J., Ji Q. (2019). Comparing the microbial community in four stomach of dairy cattle, yellow cattle and three yak herds in qinghai-tibetan plateau. Front. Microbiol..

[53] Reis M.E., de Toledo A.F., da Silva A.P., Poczynek M., Cantor M.C., Virgínío Júnior G.F., Greco L., Bittar C.M.M. (2022). Effect of supplementation with algae β-glucans on performance, health, and blood metabolites of Holstein dairy calves. J. Dairy Sci..

[54] Zhao C., Li H., Gao C., Tian H., Guo Y., Liu G., Li Y., Liu D., Sun B. (2023). Moringa oleifera leaf polysaccharide regulates fecal microbiota and colonic transcriptome in calves. Int. J. Biol. Macromol..

[55] Amin N., Seifert J. (2021). Dynamic progression of the calf’s microbiome and its influence on host health. Comput. Struct. Biotechnol. J..

[56] Tong J., Zhang H., Yang D., Zhang Y., Xiong B., Jiang L. (2018). Illumina sequencing analysis of the ruminal microbiota in high-yield and low-yield lactating dairy cows. PLoS ONE.

[57] Furman O., Shenhav L., Sasson G., Kokou F., Honig H., Jacoby S., Hertz T., Cordero O.X., Halperin E., Mizrahi I. (2020). Stochasticity constrained by deterministic effects of diet and age drive rumen microbiome assembly dynamics. Nat. Commun..

[58] Meale S.J., Li S., Azevedo P., Derakhshani H., Plaizier J.C., Khafipour E., Steele M.A. (2016). Development of ruminal and fecal microbiomes are affected by weaning but not weaning strategy in dairy calves. Front. Microbiol..

[59] Cynober L., Boucher J.L., Vasson M.P. (1995). Arginine metabolism in mammals. J. Nutr. Biochem..

[60] Wu G., Morris S.M. (1998). Arginine metabolism: Nitric oxide and beyond. Biochem. J..

[61] Frémont L. (2000). Biological effects of resveratrol. Life Sci..

[62] Piotrowska H., Kucinska M., Murias M. (2012). Biological activity of piceatannol: Leaving the shadow of resveratrol. Mutat. Res..

[63] Dai Z.L., Wu G., Zhu W.Y. (2011). Amino acid metabolism in intestinal bacteria: Links between gut ecology and host health. Front. Biosci. (Landmark Ed.).

[64] Sun C.H., Liu H.Y., Liu B., Yuan B.D., Lu C.H. (2019). Analysis of the gut microbiome of wild and captive Père David’s deer. Front. Microbiol..

[65] Li J., Zhan S., Liu X., Lin Q., Jiang J., Li X. (2018). Divergence of Fecal Microbiota and Their Associations With Host Phylogeny in Cervinae. Front. Microbiol..

[66] Jiang F., Song P., Wang H., Zhang J., Liu D., Cai Z., Gao H., Chi X., Zhang T. (2022). Comparative analysis of gut microbial composition and potential functions in captive forest and alpine musk deer. Appl. Microbiol. Biotechnol..

[67] Ortakci F., Broadbent J.R., Oberg C.J., McMahon D.J. (2015). Growth and gas production of a novel obligatory heterofermentative Cheddar cheese nonstarter *Lactobacilli* species on ribose and galactose. J. Dairy Sci..

[68] Bounaix S., Benachour A., Novel G. (1996). Presence of lactose genes and insertion sequences in plasmids of minor species of the genus *Lactococcus*. Appl. Environ. Microbiol..

[69] Warnick T.A., Methé B.A., Leschine S.B. (2002). *Clostridium phytofermentans* sp. nov., a cellulolytic mesophile from forest soil. Int. J. Syst. Evol. Microbiol..

[70] Barcenilla A., Pryde S.E., Martin J.C., Duncan S.H., Stewart C.S., Henderson C., Flint H.J. (2000). Phylogenetic relationships of butyrate-producing bacteria from the human gut. Appl. Environ. Microbiol..

