# Supplementary material for: Microbiota and Metabolite Profiles in the Feces of Juvenile Sika Deer (Cervus nippon) from Birth to Weaning
Source: Animals (Basel). 2024 Jan 29;14(3):432. doi: 10.3390/ani14030432 (PMC10854736; doi:10.3390/ani14030432)
Supplement: Supplementary file 1 [file animals-14-00432-s001.zip › animals-2758502-supplementary.pdf]

Table S1. The significantly different bacterial genera (relative abundances) in the feces of juvenile sika deer among different age-groups.

Genera	Average relative abundances			SEM	*P* value
	D1	D42	D70		
Phocaeicola	0.17^{b}	2.04^{ab}	2.62^{a}	0.004	0.017
Alloprevotella	0.18^{b}	2.39^{a}	0.27^{ab}	0.005	0.044
Alistipes	0.28^{b}	1.80^{ab}	2.57^{a}	0.003	0.014
Rikenellaceae RC9	0.74^{Bb}	4.40^{ABab}	6.51^{Aa}	0.008	0.005
Bacillus	13.10	0.50	0.33	0.030	0.048
Lactobacillus	4.72^{Aa}	0.48^{ABab}	0.01^{Bb}	0.010	0.007
Lactococcus	9.70^{a}	0.33^{ab}	0.23^{b}	0.022	0.033
Christensenellaceae R-7	0.63^{Bb}	3.78^{ABab}	4.20^{Aa}	0.005	0.007
Lachnoclostridium	3.55^{a}	0.01^{b}	0.02^{ab}	0.006	0.015
Lachnospiraceae NK4A136	0.66^{Bb}	3.77^{Aa}	1.75^{ABab}	0.004	0.011
Ruminococcaceae Other	0.47^{Bb}	2.59^{ABab}	3.67^{Aa}	0.004	0.011
Ruminococcaceae UCG-005	2.20^{Bb}	15.35^{ABab}	18.23^{Aa}	0.021	0.007
Ruminococcaceae UCG-010	0.62^{b}	5.11^{a}	6.59^{a}	0.008	0.008
Ruminococcaceae UCG-014	0.28^{b}	2.60^{a}	1.21^{ab}	0.005	0.023
Eubacterium coprostanoligenes group	0.86^{b}	4.30^{a}	3.19^{a}	0.005	0.013
Breznakia	0.00	0.01	0.01	0.000	0.042
Fusobacterium	4.47^{Aa}	0.00^{Bb}	0.00^{ABb}	0.010	0.003

SEM Standard error of the mean. a, b, c, A, B, C values within a row, different letters denote differences among different groups ($P < 0.05$, $P < 0.01$).

Table S2. The classification and proportion of identified metabolites.

Metabolites	Number	Proportions
Fatty acids	13	9.49
Amino acids	30	21.90
Amines	5	3.65
Organic acids	3	2.19
Purines	7	5.11
Pyrimidines	3	2.19
Sugars	20	14.60
Sugar alcohols	6	4.38

Table S3. Metabolic pathway enrichment analysis based on shared metabolites among the three age-groups.

Metabolites	Concentrations			Fold change		
	D1	D42	D70	D1vsD42	D1vsD70	D42vsD70
Myo-inositol	1.287	0.818	0.889	1.574	1.448	0.919
Myristic acid	0.759	0.968	0.809	0.784	0.938	1.197
Urea	0.539	0.588	0.278	0.917	1.936	2.111
Glycerol	0.166	0.193	0.276	0.860	0.601	0.699
Heptadecanoic acid	1.612	1.034	0.182	1.560	8.860	5.680
Lauric acid	0.136	0.245	0.171	0.554	0.792	1.430
Alanine	0.374	0.306	0.169	1.222	2.216	1.813
Oxamic acid	0.106	0.081	0.162	1.317	0.654	0.497
Putrescine	1.648	0.853	0.139	1.933	11.821	6.117

Lignoceric acid	0.168	0.148	0.137	1.136	1.225	1.079
Behenic acid	0.277	0.184	0.098	1.506	2.829	1.878
N-acetyl-D-galactosamine	0.099	0.085	0.088	1.158	1.117	0.965
2,6-diaminopimelic acid	0.033	0.076	0.081	0.439	0.414	0.943
Arachidonic acid	0.053	0.026	0.071	2.049	0.748	0.365
Hypoxanthine	0.098	0.029	0.065	3.352	1.505	0.449
Tyrosine	0.050	0.095	0.060	0.527	0.838	1.591
Palmitoleic acid	0.037	0.026	0.049	1.443	0.753	0.522
3-hydroxypropionic acid	0.022	0.018	0.046	1.209	0.486	0.402
Glutaric acid	0.008	0.023	0.043	0.372	0.198	0.533
Oxalic acid	0.050	0.040	0.042	1.252	1.201	0.959
Glycolic acid	0.022	0.032	0.035	0.677	0.631	0.932
2'-deoxyguanosine	0.018	0.011	0.033	1.608	0.531	0.331
3,6-anhydro-D-galactose	0.029	0.026	0.032	1.122	0.910	0.811
Arachidic acid	0.075	0.050	0.032	1.517	2.361	1.557
D-(glycero1 1-phosphate)	0.028	0.023	0.029	1.221	0.975	0.798
2-monoolein	0.029	0.018	0.027	1.620	1.084	0.669
Fucose	0.025	0.031	0.027	0.827	0.955	1.155
L-cysteine	0.028	0.015	0.025	1.934	1.132	0.586
D-galacturonic acid	0.054	0.031	0.024	1.750	2.198	1.256
3-hydroxybutyric acid	0.012	0.012	0.024	1.024	0.519	0.506
6-phosphogluconic acid	0.013	0.008	0.022	1.583	0.574	0.363
Thymine	0.013	0.015	0.021	0.849	0.606	0.714
Lactamide	0.016	0.026	0.020	0.625	0.790	1.264
Lactate	0.259	0.387	0.019	0.668	13.978	20.916
Threonine	0.026	0.017	0.018	1.537	1.414	0.920
Serine	0.029	0.015	0.018	1.885	1.578	0.837
Glutamic acid	0.026	0.014	0.016	1.845	1.618	0.877
Pyruvic acid	0.009	0.013	0.016	0.671	0.539	0.803
Maleic acid	0.008	0.008	0.016	0.929	0.486	0.524
Phenylalanine	0.023	0.009	0.015	2.566	1.591	0.620
2,3-dihydroxypyridine	0.010	0.008	0.014	1.182	0.729	0.617
Tetrahydrocorticosterone	0.034	0.020	0.012	1.674	2.940	1.756
Digitoxose	0.014	0.007	0.012	2.212	1.252	0.566
Beta-alanine	0.012	0.033	0.010	0.350	1.129	3.229
Galactonic acid	0.008	0.007	0.008	1.116	1.011	0.907
Pantothenic acid	0.004	0.002	0.008	1.565	0.476	0.304
O-methylthreonine	0.021	0.014	0.008	1.504	2.691	1.790
Fumaric acid	0.004	0.006	0.008	0.613	0.444	0.725
Nicotianamine	0.008	0.007	0.008	1.218	1.038	0.852
D-glyceric acid	0.003	0.003	0.008	1.197	0.440	0.368
Biotin	0.008	0.008	0.008	1.084	1.077	0.993
Diglycerol	0.008	0.011	0.007	0.758	1.124	1.484
Alpha-ketoglutaric acid	0.001	0.007	0.008	0.175	0.145	0.828
N-carbamylglutamate	0.004	0.002	0.006	1.944	0.641	0.330
Pelargonic acid	0.004	0.005	0.006	0.773	0.663	0.857
Galactinol	0.021	0.023	0.006	0.944	3.583	3.796
21-hydroxypregnenolone	0.011	0.005	0.006	2.497	1.982	0.794
Methyl phosphate	0.003	0.003	0.006	1.095	0.566	0.517
Erythrose	0.008	0.008	0.005	1.084	1.687	1.556

20alpha-hydroxycholesterol	0.009	0.007	0.004	1.223	1.962	1.605
Cerotinic acid	0.003	0.034	0.004	0.077	0.607	7.873
Aminomalonic acid	0.003	0.003	0.003	0.868	0.849	0.978
Inosine	0.018	0.019	0.003	0.942	6.397	6.789
O-acetylserine	0.002	0.002	0.003	1.389	0.844	0.608
Threonic acid	0.002	0.002	0.002	1.035	0.749	0.724
Threitol	0.205	0.194	0.002	1.055	121.454	115.091
Maleamate	0.002	0.002	0.002	1.194	1.248	1.045
